# To the Editors of the Medical and Physical Journal

**Published:** 1810-05

**Authors:** E. L. Knowles

**Affiliations:** Newmarket


### *To the Editors of the Medical and Physical Journal.*

GENTLEMEN,

A Disease which, I apprehend, is not attributed peculiarly to the British climate is the Plica Polonica. Being a few days ago in the shop of Mr. Rogers, printer, of this town, he presented to me for inspection a plica 9 feet long, which is now in his possession. It instantly struck me, that a malady of this nature, not peculiar to our country, existing under the influence of British atmosphere, was a most singular instance; it attracted my attention, and induced me to investigate into the particulars of the case.

In the year 1796, Mrs. Clarke, who lived in the Rectory House, Great Saxham, Suffolk, said that her mother, who was the subject of this case, had an elf-lock.* After it grew to the above length, it was accidentally trod upon and detached from the head, without producing any constitutional affection.

It seems that the excellent observations on plica in your Journal for March, by M. de la Fontaine, are substantiated by experience; he says, that it is dangerous to cut a true plica in its first stage, and when adhering to the head; we must see new sound hairs springing up, which push it downwards, before we can execute this operation with any beneficial result.

I presume that the disease which existed upon the subject in question, must have gone thro' its various changes previous to this accident, or else it would have been productive of ill consequences, epilepsy, &c. as is stated in M. Theder's letter to the author on the above subject. It appears by the information I obtained, that this plica was perfectly distinct from the sound hairs, as is described by M. de la Fontaine, when in a fit state for extirpation; but what is singular, it re-appeared, and grew to the length of 3 feet, at which time she was affected with the variola, which terminated fatally. The plica did not seem sensible to the touch, as she was in the habit of running a comb thro' it to keep it together, and she used to consider it as a great ornament. Such is the fact, that this disease has existed in this neighbourhood; whether it originated from contagion or neglect of cleanliness, I was not able to learn.

*Newmarket, April* 10, 1810. I am, &c. E. L. KNOWLES.

P.S. Does a spurious plica ever grow to the above length?

---

* I understand that the peasants call it by this name.

